# Temporal trends and patterns for early- and late-onset adult liver cancer incidence vary by race/ethnicity, subsite, and histologic type in the United States from 2000 to 2019

**DOI:** 10.1007/s10552-024-01955-4

**Published:** 2025-01-09

**Authors:** Mei-Chin Hsieh, Kendra L. Ratnapradipa, Laura Rozek, Shengdi Wen, Yu-Wen Chiu, Edward S. Peters

**Affiliations:** 1 Epidemiology and Population Health Program, School of Public Health, Louisiana State University Health Sciences Center, 2020 Gravier St., 3Rd Floor, , New Orleans, LA 70112 USA; 2 Louisiana Tumor Registry, School of Public Health, Louisiana State University Health Sciences Center, New Orleans, LA 70112 USA; 3 Epidemiology Department, College of Public Health, University of Nebraska Medical Center, Omaha, NE 68198 USA; 4 Oncology Academic Department, Georgetown University Medical Center, Washington, DC 20057 USA; 5 Cancer Prevention and Control Program for Georgetown-Lombardi Comprehensive Cancer Center, Georgetown University, Washington, DC 20057 USA

**Keywords:** Liver cancer, Intrahepatic bile duct cancer, Trend analysis, Hepatocellular carcinoma (HCC), Cholangiocarcinoma (ICC)

## Abstract

**Purpose:**

To examine incidence trends and patterns for early- and late-onset liver cancer.

**Methods:**

Liver and intrahepatic bile duct (IBD) cancers diagnosed between 2000 and 2019 were acquired from 22 SEER registries. Variables included early-onset (20–49) vs. late-onset (50+), anatomic subsite, histologic type (hepatocellular carcinoma [HCC] and IBD cholangiocarcinoma [ICC]), sex, and race/ethnicity. Age-standardized incidence rates were calculated using SEER*Stat. Jointpoint regression analysis was employed to estimate the annual percent change (APC) and the average APC (AAPC) with pairwise comparisons for trend by sex and by race/ethnicity stratified by age and subsite.

**Results:**

Liver cancer incidence decreased among early-onset (AAPC [95% CI] − 2.39 [− 2.74, − 2.07]) but increased among late-onset patients (2.85 [2.71, 3.01]), primarily driven by HCC (3.60 [3.50, 3.71]). IBD incidence increased for both ages with ICC incidence annually increasing 7.92% (6.84, 9.26) for early-onset and 6.32% (5.46, 8.86) for late-onset patients. Early-onset liver cancer displayed comparable trends across racial/ethnic groups; however, late-onset liver cancer showed more variation, particularly among American Indian/Alaska Native/Asian Pacific Islander (AI/AN/API) populations, which experienced a significant decrease in incidence, thereby narrowing the gap with other racial/ethnic groups. For IBD, an identical pattern of early-onset IBD among non-Hispanic Blacks (NHBs) compared to Hispanics was showed with coincidence test *p* = 0.1522, and a parallel pattern was observed among late-onset patients for both sexes (*p* = 0.5087).

**Conclusion:**

Late-onset HCC continues to rise, except for NHB and AI/AN/API, where incidence rates have started to decrease over the past 4–5 years. Early and late-onset ICC incidence continues to increase across all racial/ethnic groups.

**Supplementary Information:**

The online version contains supplementary material available at 10.1007/s10552-024-01955-4.

## Introduction

Globally, the number of newly diagnosed liver cancer cases per year is predicted to increase by 55% between 2020 and 2040, from 905,700 in 2020 to a projected 1.4 million people diagnosed in 2040 [1]. Although Asian countries had the highest liver cancer incidence rates, specific country-level rates in Asia have decreased in recent decades [2]. In contrast, the United States (US) showed a steadily rising incidence rate of liver cancer, with a 3.4% increase annually between 1975 and 2017 [2, 3]. In 2024, 41,630 newly diagnosed liver cancer cases and 29,840 liver cancer deaths are predicted to occur in the US [4].

Despite the incidence of overall cancer being considerably higher in older adults compared to younger adults, recently, an increasing incidence of early-onset cancers, defined as cancers diagnosed in adults < 50 years, has attracted increasing attention, especially in the US. Recent studies reported that the cancer incidence of various organs has increased among younger adults [5–12] with gastrointestinal cancers having the fastest increasing incidence rates [8]. Worldwide, early-onset liver cancer incidence rates showed a sharp decrease during the past three decades [7]. However, in the US, the greatest increase in liver cancer incidence rates were observed in adults aged 20–49 for both males (2.7%) and females (3.8%) from 2011 to 2015 [5].

Studies that assess the trends in liver cancer incidence for early- and late-onset patients to date have not been evaluated by anatomic subsite and histologic type. To fill this gap, our primary aim was to examine the incidence trends for early- and late-onset liver cancer by liver subsite, histologic type, as well as by sex, and race/ethnicity using a large database from the Surveillance, Epidemiology, and End Results (SEER) Program [13]. Our second aim was to conduct pairwise comparisons to evaluate the trend patterns for sex and race/ethnicity stratified by age group and liver subsite. We hypothesized that the incidence trends and patterns of liver cancer would differ between early- and late-onset individuals as well as vary by liver anatomic subsite, histologic type, sex, and race/ethnicity.

## Materials and methods

### Data source and data collection

Data on liver and intrahepatic bile duct (IBD) cancers diagnosed in 2000–2019 were obtained from 22 population-based SEER cancer registries, covering approximately 48.0% of the US population [13]. SEER is a program supported by the National Cancer Institute (NCI) and provides a comprehensive source of population-based cancer information with complete and high-quality data collected by the SEER registries. The SEER 22 registries’ data include 15 states (California [4 cancer registries], Connecticut, Georgia [3 cancer registries], Hawaii, Idaho, Illinois, Iowa, Kentucky, Louisiana, Massachusetts, New Jersey, New Mexico, New York, Utah, and Texas), Alaska Natives, and Seattle area in the state of Washington [14]. We used SEER Research Plus Limited-Field Data with Delay-Adjustment for trend analysis, which was adjusted for the delay reporting data from registries [15].

We used the SEER site recode, which is based on the International Classification of Disease for Oncology, 3rd Edition (ICD-O-3) topography and histology codes to category type of cancer as liver (C22.0) and IBD (C22.1) cases [16]. Histologic codes 9050–9055, 9140, and 9590–9993 were excluded. Patients with malignant liver or IBD cancer diagnosed at age 20 or older were included in this study.

### Variables

We categorized the histologic type of liver cancer as hepatocellular carcinoma (HCC) or non-HCC, and the type of IBD as cholangiocarcinoma (ICC) or non-ICC. HCC included histology codes 8170–8180 and ICC included histology code 8160. We categorized age into early-onset (age 20–49) and late-onset (age ≥ 50). Race/ethnicity included non-Hispanic white (NHW), non-Hispanic black (NHB), Hispanic (any race), and non-Hispanic American Indian/Alaska Native/Asian and Pacific Islander (AI/AN/API). The SEER summary stage 2000 (SS2000) was used to categorize cancer stage for liver and IBD cancer. The early stage included localized disease and the late-stage included regional and distant disease.

## Statistical analysis

Descriptive statistics on patient demographics and liver anatomic subsites by early- and late-onset age group were presented. Age-standardized incidence rates (ASIRs) were calculated by diagnosis year, sex, race/ethnicity, age group, anatomic site, and histologic type. The ASIRs were age-adjusted using 19 age groups to the 2000 US standard population and expressed per 100,000 person-years.

To examine changes in the incidence rate of liver cancer over time, we estimated the annual percent change (APC) and average annual percent change (AAPC) of incidence rates by age group, sex, race/ethnicity, anatomic site, and histologic type. We used Joinpoint regression model to test whether a change of ASIRs in trend was statistically significant, which was based on the Monte Carlo Permutation method to determine the simplest jointpoint model that best fits the time trend of liver and IBD cancer incidence rates [17]. Additionally, we used pairwise comparison (comparability test) to compare the sex- and race/ethnicity-specific trends stratified by age group and subsite for parallelism (test of parallelism) and identicalness (test of coincidence) [26]. Statistically significant results (*p* < 0.05) indicated a difference between the estimated slopes of the two independent comparison groups. The COVID-19 pandemic altered healthcare practices, impacted the availability of public healthcare services, and caused the public to be more hesitant about interacting with healthcare providers. This triggered delays and reductions in cancer screening and diagnosis in 2020 and possibly into 2021 [18, 19]. Therefore, we limited the trend analyses to liver and IBD cases diagnosed in 2000–2019.

Incidence rates were estimated using the SEER*Stat version 8.4.3 [20, 21]. We used Jointpoint trend analysis software version 5.1.0 (NCI, Bethesda, MD, USA) to conduct trend analyses [22]. The APCs, AAPCs, and their corresponding 95% confidence intervals (CIs) were computed. All tests of statistical significance for comparisons were set at a *p*-value of < 0.05.

This study involving human subjects was reviewed and approved by the institutional review board of the Louisiana State University Health Sciences Center—New Orleans. We use SEER public research data, so written informed consent for participation was not required for this study in accordance with national legislation and institutional requirements.

## Results

A total of 257,614 eligible liver cancer cases diagnosed in 2000–2019 were included in this study. Approximately 61.8% were microscopically confirmed and 25.3% were diagnosed solely by radiography. Early- and late-onset patients had the same distribution of liver subsite, about 88% occurred in liver and 12% in IBD area (Table 1). Males had a higher ASIR of both liver and IBD cancers compared to females for early- and late-onset diagnosis, ASIR (95% CI) 2.46 (2.42, 2.50) vs, 0.82 (0.79, 0.84) and 42.17 (41.96, 42.37) vs. 14.52 (14.41, 14.62), respectively. Sixty percent of early-onset patients were not NHW compared to only 45.7% in late-onset patients (Table 1). AI/AN/API had the highest early-onset liver/IBD incidence rate with ASIR 3.31 (3.21, 3.42) and Hispanic had the highest late-onset liver/IBD incidence rate with ASIR 47.39 (46.96, 47.83).

**Table 1:** Characteristics of liver cancer patients and incidence rates by age group, SEER 2000–2019

	20–49 years		≥ 50 years	
	*n* (%)	ASIR (95% CI)^a,b^	*n* (%)	ASIR (95% CI)^a,b^
*Liver subsite*				
Liver	17,333 (87.5)	1.43 (1.41, 1.45)	208,787 (87.8)	23.78 (23.68, 23.88)
IBD	2,470 (12.5)	0.21 (0.20, 0.21)	29,024 (12.2)	3.42 (3.38, 3.48)
*Sex*				
Male	14,860 (75.0)	2.46 (2.42, 2.50)	16,9019 (71.1)	42.17 (41.96, 42.37)
Female	4,943 (25.0)	0.82 (0.79, 0.84)	68,792 (28.9)	14.52 (14.41, 14.62)
Race/ethnicity				
NHW	7,891 (39.8)	1.13 (1.11, 1.16)	129,080 (54.3)	21.02 (20.90, 21.13)
NHB	2,951 (14.9)	2.06 (1.99, 2.14)	29,231 (12.3)	33.33 (32.93, 33.72)
Hispanic	5,209 (26.3)	2.04 (1.98, 2.10)	50,586 (21.3)	47.39 (46.96, 47.83)
AI/AN/API	3,691 (18.6)	3.31 (3.21, 3.42)	28,431 (12.0)	44.52 (43.99, 45.06)

*SEER* surveillance, epidemiology, and end results, *IBD* intrahepatic bile duct, *NHW* non-Hispanic white, *NHB* non-Hispanic black, *AI/AN/API*, AI/AN/API, American Indian/Alaska Native/Asian Pacific Islander, *ASIR* age-standardized incidence rate

^a^Incidence rates adjusted to 2000 US standard population

^b^Rates are per 100,000 population

**Fig. 1:**
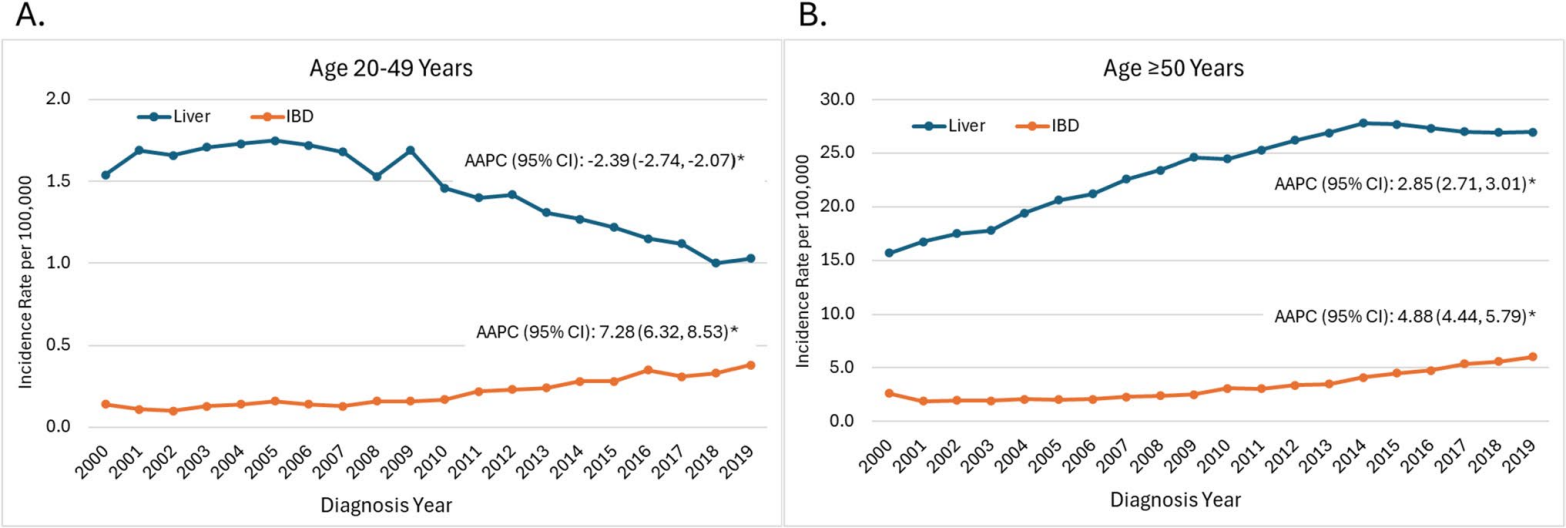
Trend of age-standardized incidence rates by age group and liver anatomic subtype, SEER 2000–2019. *SEER* Surveillance, epidemiology, and end results, *IBD* intrahepatic bile duct; *AAPC* average annual percent change

**Fig. 2:**
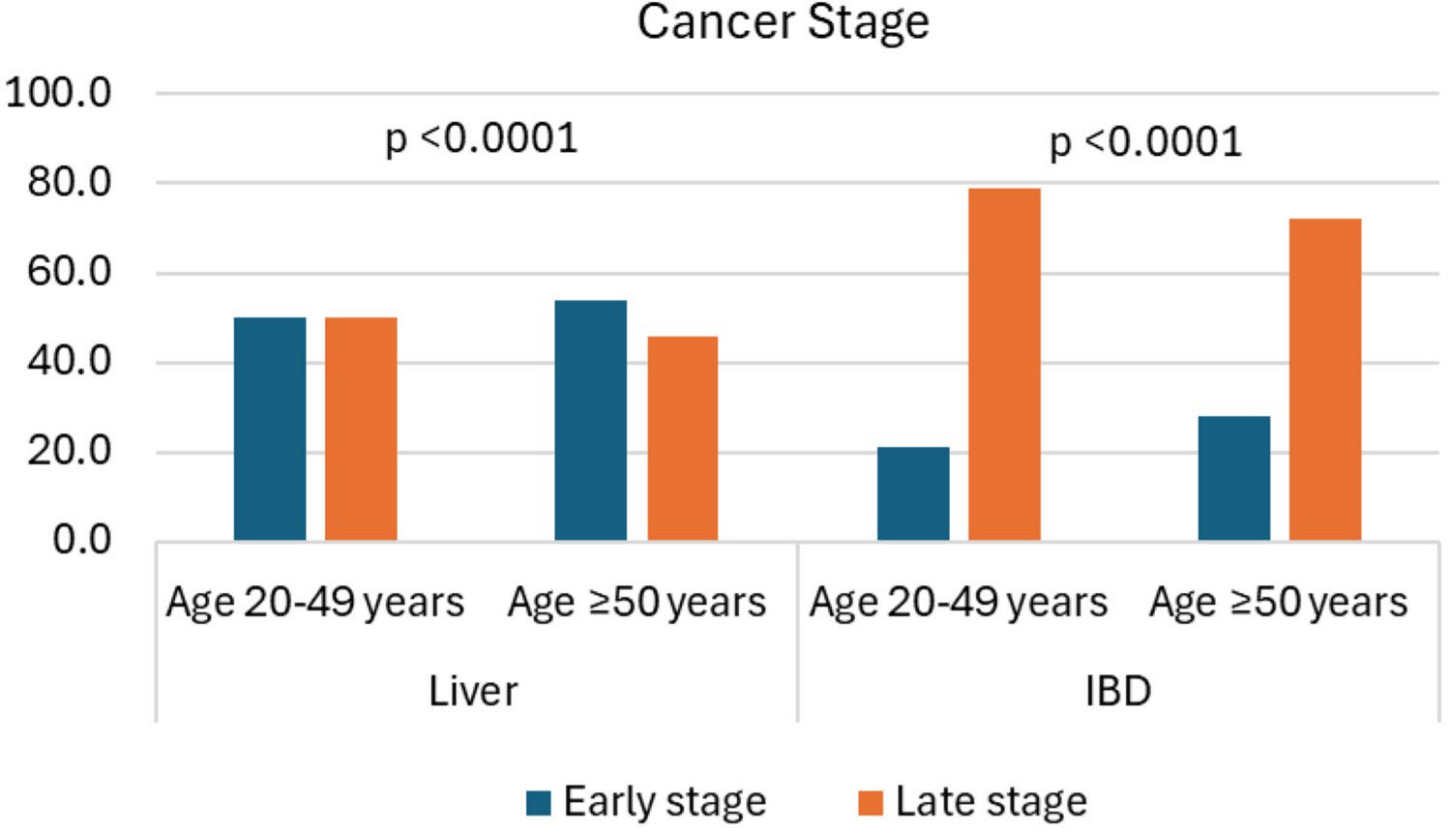
Distribution of cancer stage by age group and liver anatomic subtype, SEER 2000–2019. *SEER* Surveillance, epidemiology, and end results, *IBD* intrahepatic bile duct, early stage includes localized disease, and late-stage includes regional and distant disease, *Statistically significant at *p* <0.05.

Figure 1 shows the ASIR trends between 2000 and 2019 by liver subsite and age group. The trend of IBD cancer incidence rates increased gradually for both age groups. However, the trend for liver cancer incidence rates showed an inverse relationship, with a decreasing trend observed among the early-onset group and an increasing trend in late-onset group. The incidence rate of liver cancer among early-onset patients decreased over time between 2000 and 2019 with an AAPC (95% CI) − 2.39% (− 2.74, − 2.07). Conversely, the incidence rate of IBD increased 7.28% (6.32, 8.53) per year (Fig. 1A). Among late-onset patients, the incidence rate increased for both liver and IBD, AAPC 2.85% (2.71, 3.01) and 4.88% (4.44, 5.79), respectively (Fig. 1B). However, the late-onset liver cancer incidence trend slightly declined between 2015 and 2018. Furthermore, early-onset liver cancer patients had a higher percentage of late-stage when compared to late-onset patients, 49.9% vs 46.0% for liver and 78.6% vs. 71.2% for IBD (Fig. 2).

The liver cancer incidence trends by histologic type and age group are presented in Table 2. The incidence rate of HCC among early-onset patients decreased by 2.27% (95% CI − 2.79, − 1.76) per year, and a declining trend was observed in 2006–2019 with APC − 3.84%. Conversely, late-onset HCC patients experienced an increasing trend from 2000 to 2007 (APC 6.41%) and 2007 to 2014 (APC 3.69%) and a slight decrease from 2014 to 2019 (APC − 0.35%), with an AAPC 3.60% (3.50, 3.71) between 2000 and 2019. For non-HCC, both early- and late-onset patients had decreased AAPC, − 3.21 (− 4.37, − 2.07) and − 1.10 (− 2.04. − 0.06), respectively, with early-onset patients having a substantial increase from 2000 to 2005 (APC 6.59%) and decrease from 2005 to 2019 (APC − 6.48%).

**Table 2:** Trends of age-standardized incidence rate by primary site, histologic type, and age group, SEER 2000–2019

Cancer	Age	Trend 1		Trend 2		Trend 3		AAPC (95% CI)
		Years	APC	Years	APC	Years	APC	2000–2019
*Liver*								
HCC	20–49 years	2000–2006	1.26	2006–2019	− 3.84*			− 2.27 (− 2.79, − 1.76)*
	≥ 50 years	2000–2007	6.41*	2007–2014	3.69*	2014–2019	− 0.35*	3.60 (3.50, 3.71)*
Non-HCC	20–49 years	2000–2005	6.59*	2005–2019	− 6.48*			− 3.21 (− 4.37, − 2.07)*
	≥ 50 years	2000–2008	1.15	2008–2019	− 2.71*			− 1.10 (− 2.04, − 0.06)*
*IBD*								
ICC	20–49 years	2000–2019	7.92*					7.92 (6.84, 9.26)*
	≥ 50 years	2000–2003	− 9.44	2003–2019	9.57*			6.32 (5.46, 8.86)*
Non-ICC	20–49 years	2000–2017	− 0.11	2017–2019	35.93*			3.18 (− 0.44, 7.05)
	≥ 50 years	2000–2002	− 16.71*	2002–2016	0.62	2016–2019	17.24*	1.05 (0.003, 2.54)*

Incidence rates adjusted to 2000 US standard population

*SEER* surveillance, epidemiology, and end results, *APC* annual percent change, *AAPC* average annual percent change, *HCC* hepatocellular carcinoma, *IBD* intrahepatic bile duct, *ICC* intrahepatic bile duct cholangiocarcinoma

*Statistically significant at *p* < 0.05

Overall, both early- and late-onset patients exhibited an increased trend for ICC and non-ICC incidence rates. The early-onset ICC incidence rates steadily rose from 2000 to 2019 at 7.9% (AAPC 7.87; 95% CI 6.80, 9.20) per year. However, for late-onset patients, the ICC incidence rates significantly increased between 2003 and 2019, about 9.6% per year. Two remarkable increases for non-ICC incidence rates were observed for both age groups, 35.9% increase per year in 2017–2019 among early-onset patients and 17.2% increase per year in 2016–2019 for late-onset patients.

Table 3 presents the AAPC in liver cancer incidence stratified by sex and race/ethnicity, across different age groups and histologic types. For further insights into the trends, detailed trends with APCs (95% CI) were provided in the supplementary tables: Supplement Table 1 focuses on early-onset patients, while Supplement Table 2 covers late-onset patients. Early-onset HCC and non-HCC rates decreased over time in both sexes and all racial/ethnic groups, but predominant in males and not NHW patients (Table 3). Among males, the decreased trend for early-onset HCC incidence rates was observed in 2006–2019 with APC (95% CI) − 4.62 (− 5.65, − 3.94) and for early-onset non-HCC rates occurred in 2005–2019 with APC − 6.35 (− 11.06, − 4.77) (Supplement Table 1). Overall, NHB had the highest percentage of decreasing rate for early-onset HCC (AAPC − 4.28; 95% CI − 5.52, − 3.22) and AI/AN/API had the highest decreasing rate for early-onset non-HCC (AAPC − 5.81; 95% CI − 8.35, − 3.66). Among NHW aged 20–29, a significant decreasing trend in HCC was observed from 2006 to 2019, with an APC of − 4.18 (− 7.45, − 3.22). Similarly, for non-HCC individuals in the same age group, a decrease was noted from 2005 to 2019, with an APC of − 4.69 (− 11.25, − 2.88). Among Hispanic individuals aged 20–49, the most pronounced decline in HCC incidence occurred between 2009 and 2019, with an APC of − 5.69 (95% CI: − 7.79 to − 4.56) (Supplement Table 1). In contrast with the early-onset HCC incidence trend, AAPCs of late-onset HCC incidence increased in both sexes and all racial/ethnic groups except for AI/AN/API. However, a statistically significant decrease trend of late-onset HCC for NHB was observed in 2016–2019 with APC − 5.04 (− 8.46, − 2.74) and in 2015–2019 for AI/AN/API with API − 4.49 (− 8.15, − 2.67) (Supplement Table 2). Early- and late-onset ICC rates increased in both sexes and all racial/ethnic groups. Females had higher percent of rates increase than males on both ICC and non-ICC with rates of early-onset ICC increasing by 8.9% (AAPC 8.92 [7.45, 11.21]), late-onset ICC increasing by 6.8% (AAPC 6.84 [5.92, 8.45]), early-onset non-ICC increasing by 5.6% (AAPC 5.55 [2.82, 9.36]), and late-onset non-ICC increasing by 2.7% (AAPC 2.65 [1.31, 3.94]). Among racial/ethnic groups, we observed that Hispanics had the highest increase of early-onset ICC and NHB had the highest increase of late-onset ICC, 9.1% (AAPC 9.13 [7.29, 12.30]) and 6.4% (AAPC 6.43 [4.77, 9.46[) per year, respectively (Table 3).

**Table 3:** Annual percent change (AAPC) of liver cancer incidence by sex and race/ethnicity stratified by age group and histology type, SEER 2000–2019

Age 20–49 years	HCC	Non-HCC	ICC	Non-ICC
	AAPC (95% CI)	AAPC (95% CI)	AAPC (95% CI)	AAPC (95% CI)
*Sex*				
Male	− 2.90 (− 3.44, − 2.43)*	–3.58 (− 5.50, − 1.87)*	6.12 (4.98, 7.40)*	0.21 (− 2.50, 2.84)
Female	− 0.11 (− 1.45, 1.18)	− 1.60 (− 3.27, − 0.04)*	8.92 (7.45, 11.21)*	5.55 (2.82, 9.36)*
*Race/ethnicity*				
NHW	− 2.65 (− 3.66, − 1.83)*	− 1.48 (− 4.20, 0.98)	5.82 (4.34, 8.06)*	1.39 (0.02, 2.73)*
NHB	− 4.28 (− 5.52, − 3.22)*	− 5.31 (− 11.49, − 2.37)*	7.60 (4.27, 12.89)*	NA^a^
Hispanic	− 3.39 (− 4.08, − 2.73)*	− 4.85 (− 6.59, − 3.27)*	9.13 (7.29, 12.30)*	1.77 (− 3.37, 8.57)
AI/AN/API	− 3.19 (− 4.02, − 2.26)*	− 5.81 (− 8.35, − 3.66)*	3.71 (1.29, 6.96)*	NA^a^
Age ≥ 50 years	AAPC (95% CI)	AAPC (95% CI)	AAPC (95% CI)	AAPC (95% CI)
*Sex*				
Male	3.54 (3.39, 3.71)*	− 1.07 (− 2.30, 0.18)	5.87 (5.00, 7.20)*	0.25 (− 0.81, 1.77)
Female	3.13 (2.92, 3.35)*	− 1.30 (− 2.17, − 0.41)*	6.84 (5.92, 8.45)*	2.65 (1.31, 3.94)*
*Race/ethnicity*				
NHW	3.87 (3.74, 4.04)*	− 1.19 (− 2.08, − 0.31)*	5.70 (5.04, 6.89)*	1.29 (0.26, 2.82)*
NHB	3.48 (3.15, 3.87)*	− 1.47 (− 3.59, 0.38)	6.43 (4.77, 9.46)*	2.91 (0.21, 5.26)*
Hispanic	2.87 (2.63, 3.25)*	− 0.70 (− 1.70, 0.59)	5.63 (4.50,7.66)*	− 1.34 (− 3.81, 2.44)
AI/AN/API	− 0.89 (− 1.25, − 0.43)*	− 3.64 (− 4.72, − 2.49)*	5.37 (4.23, 7.15)*	0.34 (− 1.25, 2.16)

*SEER* surveillance, epidemiology, and end results, *IBD* intrahepatic bile duct, *HCC* hepatocellular carcinoma, *ICC* intrahepatic bile duct cholangiocarcinoma, *NHW* non-Hispanic white, *NHB* non-Hispanic black, *AI/AN/API* AI/AN/API, American Indian/Alaska Native/Asian Pacific Islander

^a^Jointpoint cannot process due to zero case count for some diagnosis years

*Statistically significant at 0.05

**Fig. 3:**
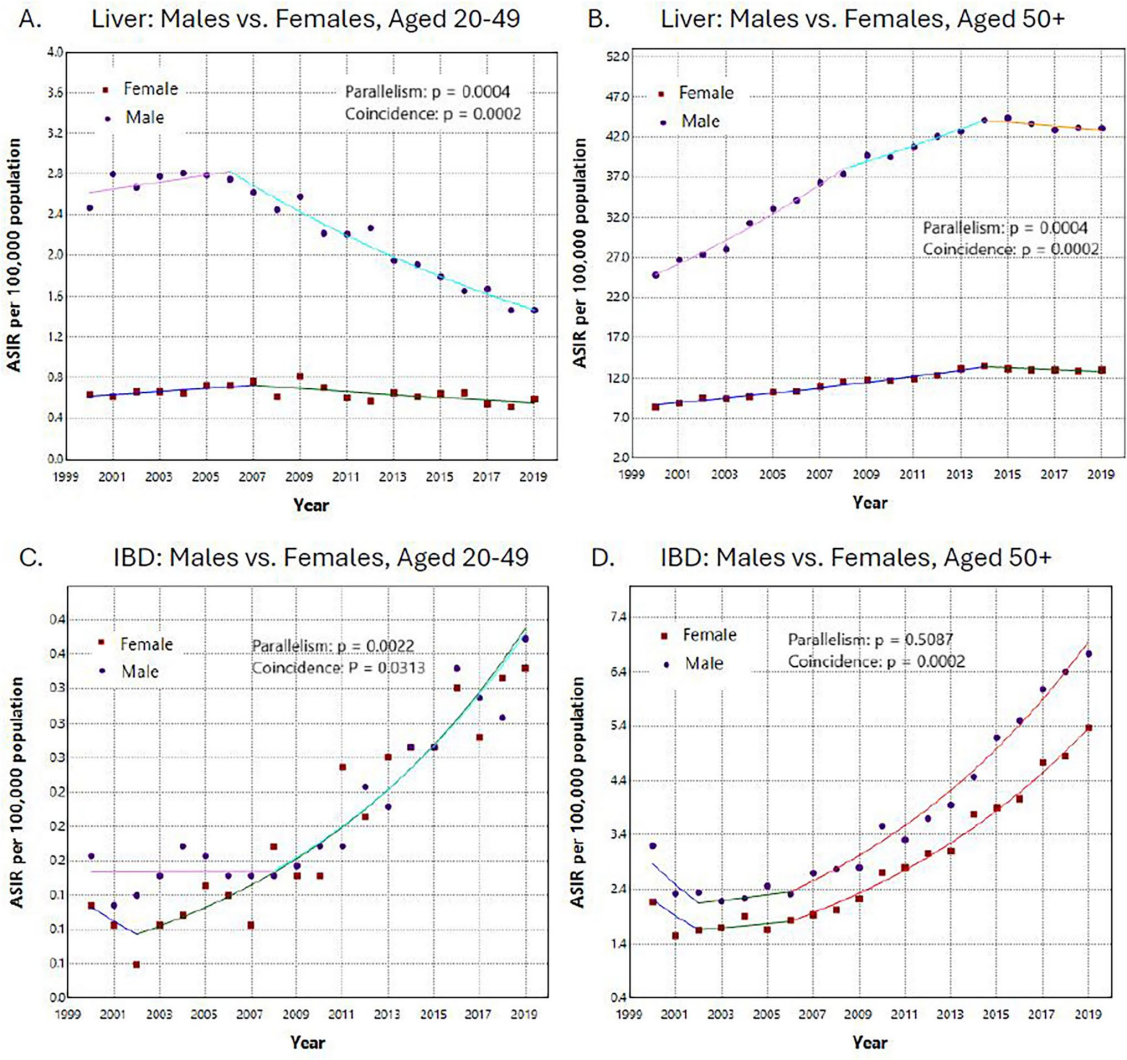
Pairwise comparisons of sex stratified by age group and liver subsite, SEER 2000–2019. *SEER* Surveillance, epidemiology, and end results, *IBD* intrahepatic bile duct

Figure 3 shows the comparisons of trend patterns by sex stratified by age group and subsite. Overall, males had higher liver cancer incidence rate than females. Among early-onset liver cancer patients, males had a sharp decrease observed in 2007–2019 and females showed slightly decreasing in 2008–2019 with non-parallel pattern (*p* = 0.0004) (Fig. 3A). In contrast, among late-onset liver cancer patients, males had a rapid increase between 2000 and 2014, and females had a gradual increase at the same time with non-parallel pattern (*p* = 0.0004) (Fig. 3B). Additionally, we observed a parallel increase pattern (*p* = 0.5087) between males and females for late-onset IBD with a dramatical increase in 2006–2019 (Fig. 3D). Although, the overall parallelism was not observed for sex among early-onset IBD patients, from 2008 to 2019 the trend lines for males and females overlapped (Fig. 3C).

Supplemental Figs. 1–4 present the pairwise comparisons of incidence trends stratified by age group, subsite, and race/ethnicity. For early-onset liver cancer (Suppl Fig. 1), parallel patterns were observed for NHW vs. Hispanic, NHB vs. Hispanic, NHB vs. AI/AN//API, and Hispanic vs. AI/AN//API with *p* > 0.05. In addition, an identical pattern was found for NHB vs. Hispanic with test for coincidence *p* = 0.1522 (Suppl Fig. 1D). Among late-onset liver patients (Suppl Fig. 2), AI/AN/API started to show a decrease rate in 2008 and the difference in incidence rate between AI/AN/API and NHB had dramatically reduced after 2015 (Suppl Fig. 2E), also the incidence rate in Hispanics surpassed that in AI/AN/API after 2009 (Suppl Fig. 2F). The incidence trend patterns of IBD for race/ethnicity pairwise comparisons were similar between early- and late-onset patients (Suppl Figs. 3, 4) with parallel increase trends observed for NHW vs. NHB, NHW vs. Hispanic, and NHB vs. Hispanic (*p* > 0.05) for both age groups.

## Discussion

Overall, the incidence of liver and IBD cancer continues to increase for individuals aged 50 years and older in the US. However, the incidence trend for individuals aged 20–49 years decreased for liver but increased for IBD. The increase in early-onset IBD (7.3% per year) is much higher than those of late-onset IBD (4.9% per year), particularly in the non-ICC incidence with about 36% increase in the 2017–2019 period for early-onset and 17.6% increase in 2016–2019 period among late-onset. This is especially concerning as approximately 80% of the IBD cases in patients aged 20–49 are diagnosed with late-stage cancers, compared to approximately 50% of liver cancers. Additionally, we found that HCC and non-HCC incidence decreased more in males than females and ICC and non-ICC incidence increased more in females than males in the early-onset group. Hispanic individuals aged 20–49 had the highest percent change in ICC incidence (9.1% per year) and AI/AN/API aged ≥ 50 showed a significantly decreased trend of HCC incidence (0.9% per year).

Previous studies reported an inconsistent trend of liver cancer incidence rates [3.5]. Ward et al. reported an increased trend of liver cancer in 2011–2015 among aged 20–49 adults [5] and Yao et al. observed a decreased trend in 2005–2017 for aged < 50 individuals [3]. However, the incidence rates and trends of their studies were estimated by either combining liver (C220) and IBD (C221) or using anatomic subsite to determine HCC and ICC without considering histology. We performed a comprehensive trend analysis for liver cancer incidence by anatomic subsite and histologic type. By dividing liver and IBD with specified histologic types, we observed declines in liver cancer incidence for both HCC (2006–2019) and non-HCC (2005–2019) among early-onset patients. The decrease in early-onset liver cancer can be partially attributed to the availability of the hepatitis B vaccine, which was introduced in the early 1980s. Yet, the incidence trends of early-onset IBD cancer showed different directions, with ICC gradually rising over time and non-ICC with a sharp increase in the 2017–2019 period, which warrants further study by anatomic subsite and histologic type. For late-onset patients, HCC shows a slightly declining trend between 2014 and 2019, and ICC cancer has had a persistent upward trend since 2003. Similar to prior studies [23–25], our study observed that males have significantly higher liver (HCC and non-HCC) and IBD (ICC and non-ICC) cancer incidence rates than females for both early- and late-onset adults, except for early-onset ICC rate. The significant differences in liver cancer incidence observed between males and females may be attributed to differences in HCV infection. In the US, the prevalence of HCV infection in males is 2.3 times that in females [26]. Compared to NHW, AI/AN/API have the highest HCC and ICC incidence rates among early-onset patients, and Hispanics have the highest late-onset HCC, non-HCC, and ICC.

Although early-onset HCC and non-HCC incidence rates have decreased since 2006, the early-onset ICC increased significantly over time in our study, and the percentage of rate increase per year among females is 2.7% higher than males. The increased ICC trends were observed in late-onset patients as well, which is consistent with Welzel’s report [27]. Furthermore, the increase of ICC incidence trends is observed for all racial/ethnic groups in both age groups with Hispanics having the highest increase on early-onset ICC incidence rate and NHBs having the highest percentage increase on late-onset ICC. Previous studies have suggested that the rising incidence of ICC in the United States is largely attributable to improved clinical distinctions between ICC and cancers of unknown primary origin [28, 29]. From 2003 to 2012, there was a notable increase in ICC cases, with an APC of 4.36% (95% CI: 3.39, 5.33). This upward trend continued from 2013 to 2017, with an APC of 10.49% (95% CI: 7.57, 13.5). In contrast, cases of unknown primary cancer exhibiting histologies consistent with cholangiocarcinoma have been on the decline since 1980 [28, 29].

Commonly known risk factors related to liver and IBD cancer include chronic viral hepatitis B (HBV) and C (HCV) infection, liver cirrhosis, metabolic dysfunction-associated fatty liver disease (MAFLD), type 2 diabetes, and modifiable risk factors comprising heavy alcohol consumption, tobacco use, and obesity [30–37]. Also, genetic instability could be a major driver in tumorigenesis and development of cancer [38]. Several somatic mutations (e.g., TERT, TP53, CTNNB1, ARID1A) related to HCC have been reported [39, 40]. However, whether these risk factors have the same impact on early- and late-onset liver/IBD cancer is unclear. Tsilimigras et al. reported that some variations of genomic features were found between early- and late-onset ICC, such as BAP1, ARID1A, and PBRM1, that were mostly mutated in late-onset ICC, are rarely mutated in the early-onset ICC patients [41]. Given that many risk factors associated with liver and IBD cancers can be mitigated through healthy lifestyle choices, further research into modifiable risk factors specific to younger populations is essential.

The strengths of this study include using high-quality data from SEER 22 registries and the adjustment for delayed reporting data. Adjusting for data delay is crucial because, in general, for cases diagnosed in the most recent years have about 4% decrease in the number of cancers submitted. The utilization of the delay-adjusted data can more precisely determine current cancer trends by adjusting the current case count to account for anticipated future corrections [42]. Additionally, we ended our analysis with 2019 data to avoid potential confounding of the trends due to the impacts of the COVID-19 pandemic on healthcare access and utilization. This triggered delays and reductions in cancer screening and diagnosis in 2020 and possibly 2021 [18, 19], which likely result longer-term implications such as decreased incidence during 2020–2021 followed by subsequent increases in more advanced stage diagnoses as regular healthcare patterns resume. In contrast to previous studies that combined liver and IBD for trends analyses, we evaluated the temporal trends by anatomic subsite and histologic type while comparing trends between early- and late-onset patients; this provides a more detailed and nuanced analysis. These data indicate that studies of early-onset cancers should be careful to disaggregate subtypes of disease to fully appreciate trends in the data. Our study has a few limitations. Although the data of the SEER 22 registries covered nearly half of the US population, it cannot be presented as the whole of the US. Another limitation of our study was the smaller sample sizes of the younger adults. Using data solely from the SEER database, we were unable to obtain the estimates for non-ICC trends among NHB and AI/AN/API individuals aged 20–49. Despite its limitations, our results provide fundamental information that can be utilized for future research.

## Conclusion

In our analysis, we observed that liver/IBD cancer incidence varied by age group, anatomic subtype, histologic type, as well as by sex and race/ethnicity. Compared to NHW individuals, other racial/ethnic individuals had higher liver incidence rates over time across age groups. Our data also indicates that the changing epidemiology of liver and IBD related cancers vary by age cohort, emphasizing the importance of disaggregating data to provide clearer insights into trends, which may inform causal studies of risk profiles. Research on biological mechanisms and pathogenic variation between early- and late-onset HCC/ICC are warranted to understand the pathway of cancer formation in different age groups, which could help to develop a standard liver cancer screening protocol with the ultimate goal to diagnose cancer in early stage in the future. Additionally, further study into potential differential impacts of known modifiable risk factors by age group, anatomic subtype, and histologic type are warranted. Although, HCV vaccine is not yet available, state policy decision makers can provide advocacy and education for the general population regarding healthier lifestyles and hygiene habits for cancer prevention, as well as screening for and treatment of HCV.

## Supplementary Information

Below is the link to the electronic supplementary material.

Supplement Figure 1. Pairwise comparisons of race/ethnicity for early-onset liver cancer, SEER 2000-2019. Abbreviation: SEER, Surveillance, Epidemiology, and End Results; NHW, non-Hispanic white; NHB, non-Hispanic black; AI/AN/API, American Indian/Alaska Native/Asian Pacific Islander.
Supplementary file1 (JPG 1605 KB)

Supplement Figure 2. Pairwise comparisons of race/ethnicity for late-onset liver cancer, SEER 2000-2019. Abbreviation: SEER, Surveillance, Epidemiology, and End Results; NHW, non-Hispanic white; NHB, non-Hispanic black; AI/AN/API, American Indian/Alaska Native/Asian Pacific Islander.
Supplementary file2 (JPG 378 KB)

Supplement Figure 3. Pairwise comparisons of race/ethnicity for early-onset intrahepatic bile duct cancer, SEER 2000-2019. Abbreviation: SEER, Surveillance, Epidemiology, and End Results; NHW, non-Hispanic white; NHB, non-Hispanic black; AI/AN/API, American Indian/Alaska Native/Asian Pacific Islander.
Supplementary file3 (JPG 327 KB)

Supplement Figure 4. Pairwise comparisons of race/ethnicity for early-onset intrahepatic bile duct cancer, SEER 2000-2019. Abbreviation: SEER, Surveillance, Epidemiology, and End Results; NHW, non-Hispanic white; NHB, non-Hispanic black; AI/AN/API, American Indian/Alaska Native/Asian Pacific Islander.
Supplementary file4 (JPG 388 KB)

Supplementary file5 (XLSX 34 KB)

## Data Availability

The data that support the findings of this study are publicly available through the NCI’s Surveillance, Epidemiology, and End Results Program (SEER). (https://seer.cancer.gov/data/).
