# Supplementary figures and images for: Temporal trends and patterns for early- and late-onset adult liver cancer incidence vary by race/ethnicity, subsite, and histologic type in the United States from 2000 to 2019
Source: Cancer Causes Control. 2025 Jan 9;36(5):551–60. doi: 10.1007/s10552-024-01955-4 (PMC11982089; doi:10.1007/s10552-024-01955-4)

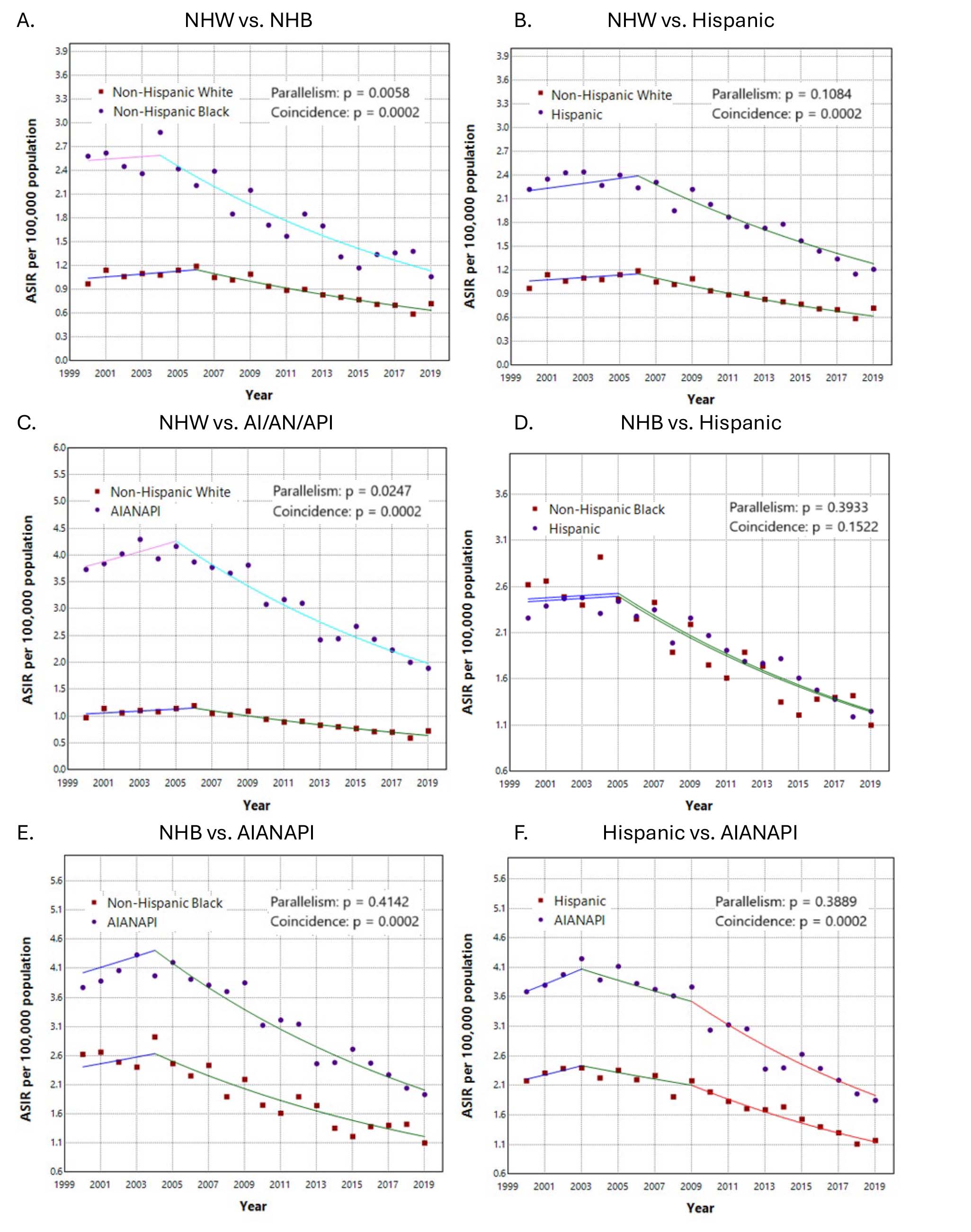

Supplement: Supplementary file 1 — Supplement Figure 1. Pairwise comparisons of race/ethnicity for early-onset liver cancer, SEER 2000-2019. Abbreviation: SEER, Surveillance, Epidemiology, and End Results; NHW, non-Hispanic white; NHB, non-Hispanic black; AI/AN/API, American Indian/Alaska Native/Asian Pacific Islander. Supplementary file1 (JPG 1605 KB) [file 10552_2024_1955_MOESM1_ESM.jpg]

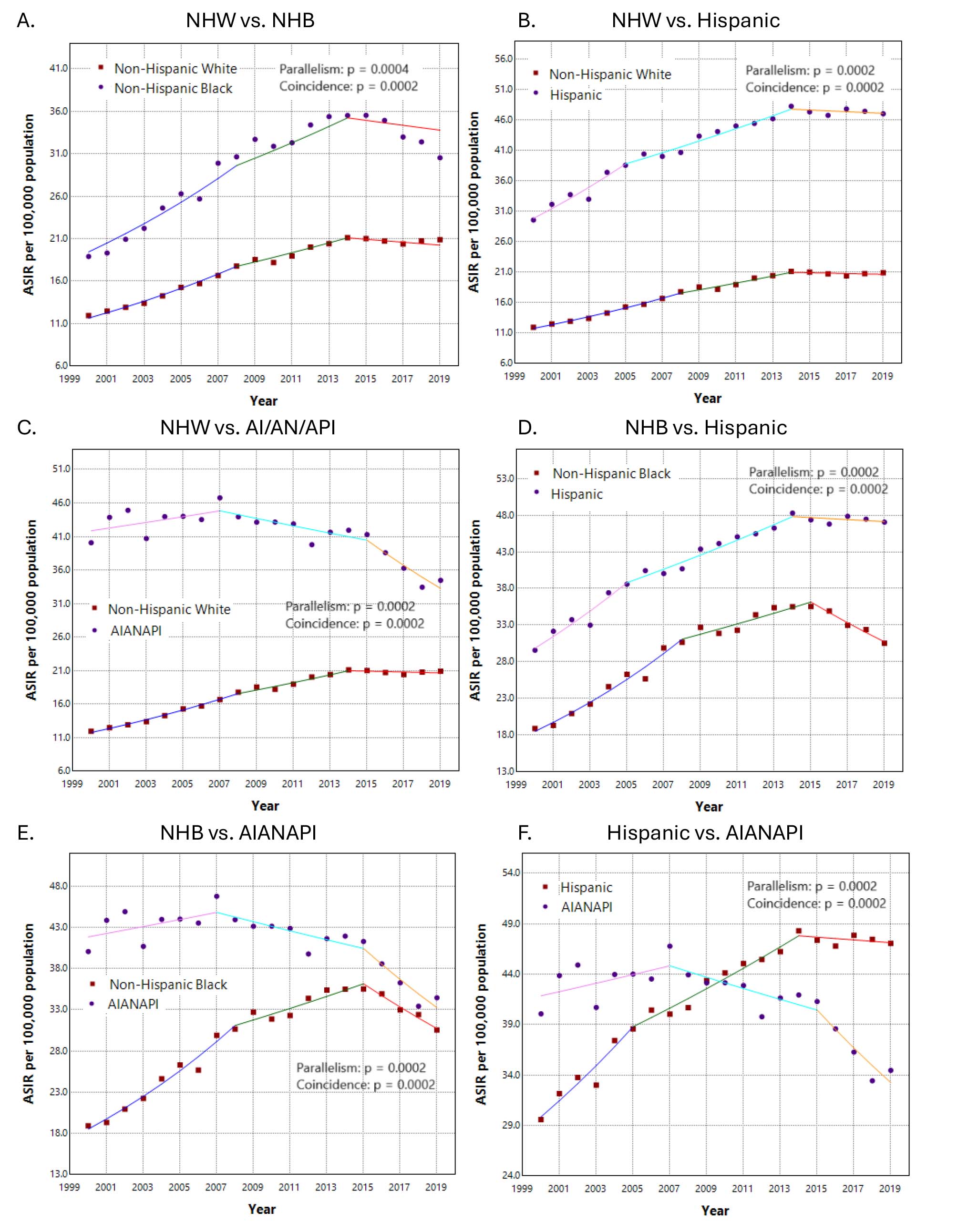

Supplement: Supplementary file 2 — Supplement Figure 2. Pairwise comparisons of race/ethnicity for late-onset liver cancer, SEER 2000-2019. Abbreviation: SEER, Surveillance, Epidemiology, and End Results; NHW, non-Hispanic white; NHB, non-Hispanic black; AI/AN/API, American Indian/Alaska Native/Asian Pacific Islander. Supplementary file2 (JPG 378 KB) [file 10552_2024_1955_MOESM2_ESM.jpg]

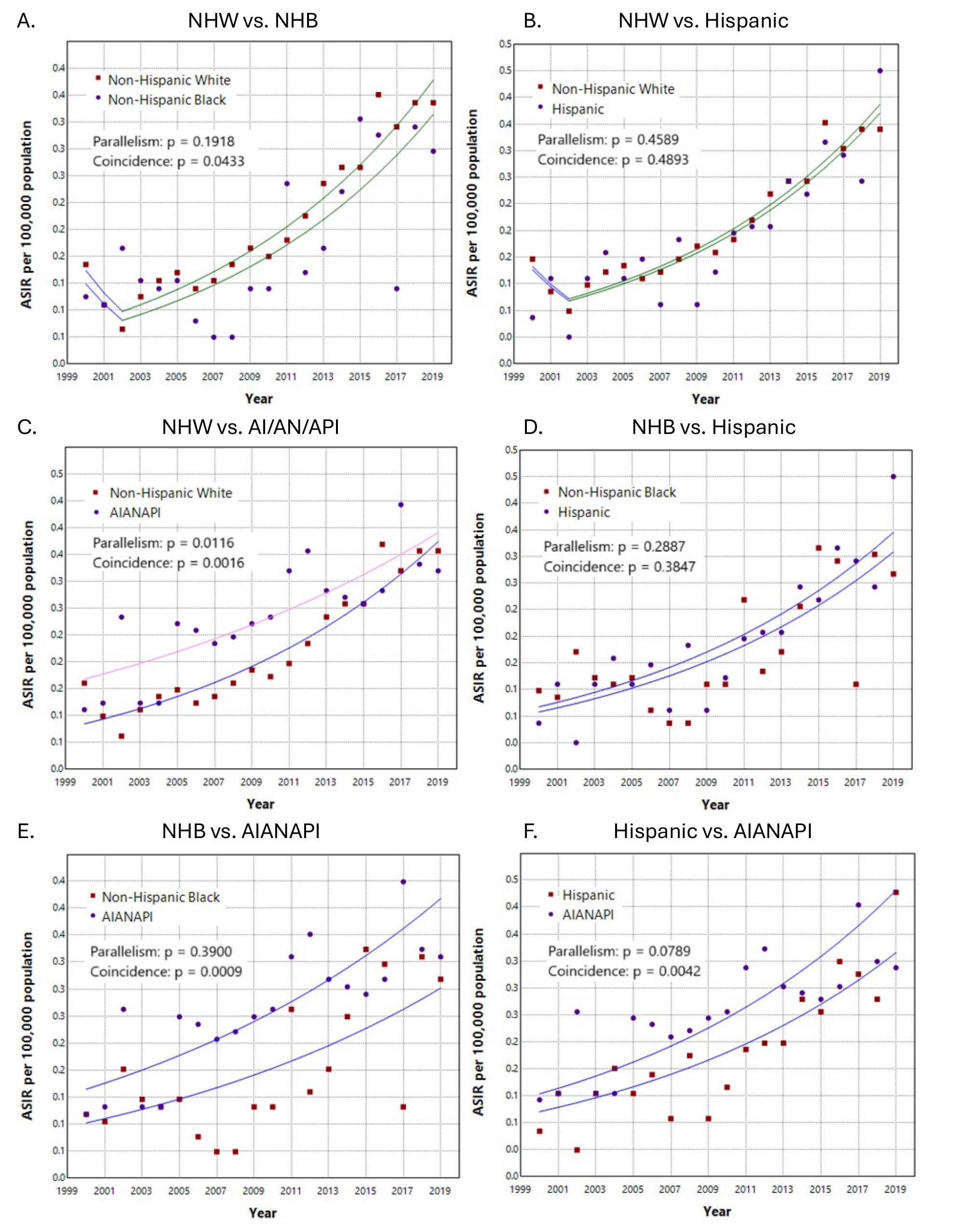

Supplement: Supplementary file 3 — Supplement Figure 3. Pairwise comparisons of race/ethnicity for early-onset intrahepatic bile duct cancer, SEER 2000-2019. Abbreviation: SEER, Surveillance, Epidemiology, and End Results; NHW, non-Hispanic white; NHB, non-Hispanic black; AI/AN/API, American Indian/Alaska Native/Asian Pacific Islander. Supplementary file3 (JPG 327 KB) [file 10552_2024_1955_MOESM3_ESM.jpg]

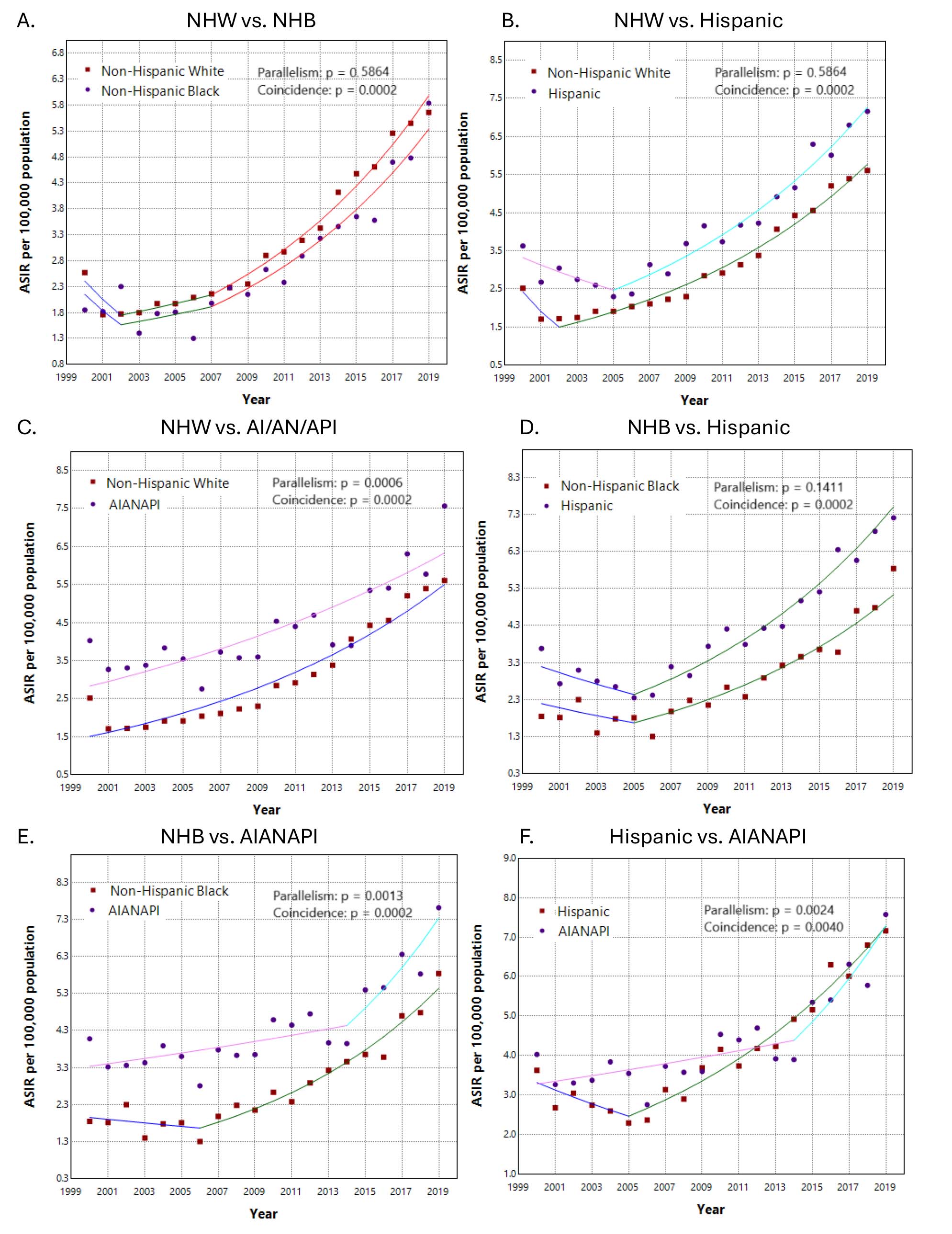

Supplement: Supplementary file 4 — Supplement Figure 4. Pairwise comparisons of race/ethnicity for early-onset intrahepatic bile duct cancer, SEER 2000-2019. Abbreviation: SEER, Surveillance, Epidemiology, and End Results; NHW, non-Hispanic white; NHB, non-Hispanic black; AI/AN/API, American Indian/Alaska Native/Asian Pacific Islander. Supplementary file4 (JPG 388 KB) [file 10552_2024_1955_MOESM4_ESM.jpg]
